# Glass Polarization Induced Drift of a Closed-Loop Micro-Accelerometer

**DOI:** 10.3390/ma11010163

**Published:** 2018-01-20

**Authors:** Wu Zhou, Jiangbo He, Huijun Yu, Bei Peng, Xiaoping He

**Affiliations:** 1 School of Mechanical and Electrical Engineering, University of Electronic Technology and Science of China, Chengdu 611731, China; yuhjuestc@126.com; 2 School of Mechanical Engineering, Xihua University, Chengdu 610039, China; chuihaol@aliyun.com; 3 Institute of Electronic Engineering, China Academy of Engineering Physics, Mianyang 621900, China; hexpiee@263.net

**Keywords:** glass polarization, silicon-on-glass, micro sensor, drift

## Abstract

The glass polarization effects were introduced in this paper to study the main cause of turn-on drift phenomenon of closed-loop micro-accelerometers. The glass substrate underneath the sensitive silicon structure underwent a polarizing process when the DC bias voltage was applied. The slow polarizing process induced an additional electrostatic field to continually drag the movable mass block from one position to another so that the sensing capacitance was changed, which led to an output drift of micro-accelerometers. This drift was indirectly tested by experiments and could be sharply reduced by a shielding layer deposited on the glass substrate because the extra electrical filed was prohibited from generating extra electrostatic forces on the movable fingers of the mass block. The experimental results indicate the average magnitude of drift decreased about 73%, from 3.69 to 0.99 mV. The conclusions proposed in this paper showed a meaningful guideline to improve the stability of micro-devices based on silicon-on-glass structures.

## 1. Introduction

Drift issue is considered as a big obstacle to developing highly-precise microelectromechanical systems (MEMS) products. Compared with the thermal drift and environmental drift, the dielectric charging induced drift had more significant importance because it exhibited diversity and complexity and appeared in almost every micro device, including dielectric materials operated under DC (Direct Current) voltage. Such a drift of micro-switches, micro-mirrors, and micro-resonators was analyzed in detail in the authors’ review literature [1]. However, the drift of inertial micro-sensors, including gyroscopes and accelerometers, was still believed to be merely related to the micro-scale thermal effects and studied extensively [2,3,4]. In fact, it does not seem reasonable to consider the thermal drift as the underlying mechanism in explaining the long-term drift phenomenon in closed-loop micro-accelerometers during the turn-on period, since the time scale of the drift, from tens of minutes to a few hours, is much longer than the preheating process, normally under a few minutes. Such a long-time drift observed in experiments might result from the dielectric charging of dielectric materials in the micro-accelerometer, because the charging effects of dielectrics, such as oxide silicon, nitride silicon, and glass involved a slow process of charge movement and took a long time to finish. The dielectric charging usually involved ion injection and internal polarization of insulation material which was used for electrical isolation or structural substrate [5,6,7,8,9], and both dielectrics and DC electrical field simultaneously existing was the necessary condition of dielectric charging [10,11]. In our studied accelerometers, the DC voltage was applied for generating feedback electrostatic forces and the dielectric materials included the glass substrate and potential oxidation layer on the sensing silicon electrodes. Therefore, the authors adopted a material measurement method, Auger electron spectroscopy (AES), to analyze the surface composition of sensing electrodes. The measurement results showed the dielectric layers on silicon electrodes are very thin, normally in the range of sub-nanometers. This layer certainly formed an additional electrical field to disturb the static balance sustained by the mechanical domain and the electrostatic domain and induced a specific level drift [12], but the drift amount and level were much smaller than that observed in experiments. 

Thus, the authors recognized the polarization effect of the glass substrate as the main contributor of the drift of capacitive closed-loop micro-accelerometers because the charges in the glass would redistribute under DC voltage. The polarization of the glass under the DC electrical field can be found in both glass films and solids and the corresponding studies were carried out from the 1960s to the 2010s [13,14,15,16,17,18,19]. This polarization could induce the reliability issue of RF (Radio Frequency) MEMS switches by forming an extra pulling force between bridge and substrate [20], and the charge movement caused by polarization could lead to the capacitance drift of micro-capacitors [21] and the angle drift of micro-mirrors [22] through changing the equivalent capacitance between two electrodes. In the micro-accelerometers, the migrated charges in glass polarization would form an electrical field to generate electrostatic forces on the silicon electrodes and alter the equilibrium of mass block due to the asymmetrical layout of bulk-machined structures. Thus, the new equilibrium state would change the differential capacitance and ultimately shift the output voltage of the capacitive micro-accelerometer. The detailed polarization effects were given in Section 3, which was following the introduction of micro-accelerometers in Section 2 and followed by the experiments and conclusions in Section 4 and Section 5, respectively.

## 2. Materials and Structure

**Figure 1:**
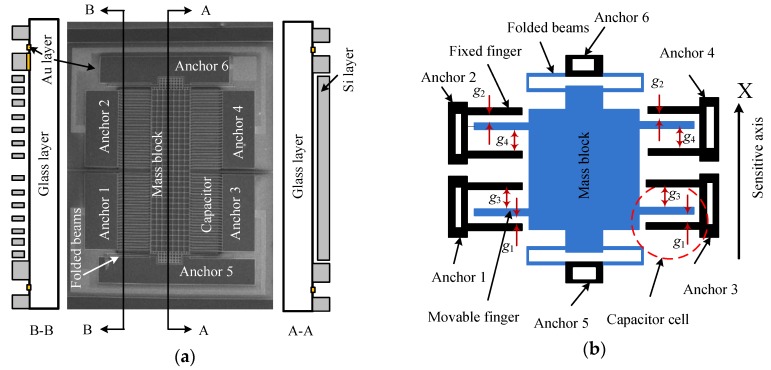
Sensor chip of the micro-accelerometer: (**a**) bi-layer structure of the micro-accelerometer; and (**b**) the simplified model of the sensor structure.

The sensitive component of a micro-accelerometer is a bi-layer structure bonding the separately-fabricated silicon chip and the glass substrate (Figure 1a). The former is fabricated on a silicon wafer by deep reaction ion etching (DRIE) and KOH wet etching technologies. The sensor chip consists of a mass block supported by two folded beams and the sensing capacitors which are constructed by the fixed electrodes on anchors and the movable electrodes on the mass block. The glass substrate is a rectangle borosilicate glass patterned with gold wires by a sputtering process. The details of the structure and fabrication process can be found in [23,24,25].

**Figure 2:**
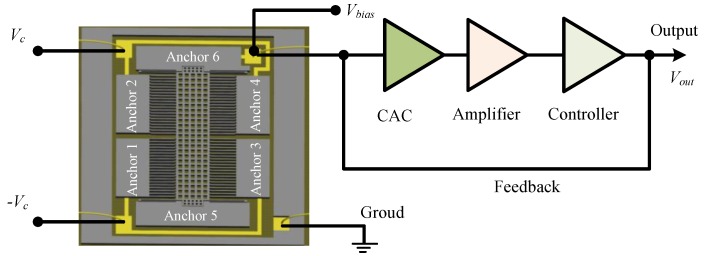
Sensing model of the accelerometer.

The sensing capacitor includes a group of capacitor cells in parallel, and its simplified model with a single cell is extracted to represent the whole structure for a clear description (Figure 1b). The electrodes on anchors 1 and 3 are the bottom electrode while those on anchors 2 and 4 are the top electrode, and the fingers on mass block are the movable electrode. The sensing model of the closed-loop accelerometer can be represented by the diagram of Figure 2. The bias voltage, *V_bias_*, contains components of DC (direct current) and AC (alternating current) parts. The former is used to generate the feedback force together with the feedback voltage, while the latter is the carrier voltage for the capacitance-voltage conversion. The input acceleration is measured by the sensitive structure converting it into the change of differential capacitance of sensing capacitors, which is sensed by the circuit connecting to mass block on anchor 6 and converted into DC voltage, *V_out_*, through charge amplifier circuit (CAC), voltage amplifier and PID (Proportional-Integral-Derivative) controller. The relationship between capacitance and output voltage can be expressed as:

(1) $$V_{out}=\frac{\left(C_{t}-C_{b}\right)A_{c}V_{a}}{C_{t}+C_{b}}$$

where *V_out_* is the output voltage of accelerometer; *V_a_* is the amplitude of AC carrier signal; *A_c_* is the amplification coefficient of sensing circuit; *C_t_* and *C_b_* are the capacitances of top capacitor formed by top electrode and movable electrode and the bottom capacitor formed by the bottom electrode and movable electrode, respectively, which can be expressed as:

(2) $$C_{t}=\frac{\varepsilon_{0}\varepsilon_{r}A}{g_{2}-x}+\frac{\varepsilon_{0}\varepsilon_{r}A}{g_{4}+x},\;C_{b}=\frac{\varepsilon_{0}\varepsilon_{r}A}{g_{1}+x}+\frac{\varepsilon_{0}\varepsilon_{r}A}{g_{3}-x}$$

where *ε*_0_ is the permittivity of vacuum; *ε_r_* is the relative dielectric constant of air, *ε_r_* = 1; *A* is the effective area of all the over lapped electrodes; *x* is the mass displacement; *g*_2_ and *g*_1_ are the initial narrow gaps of top and bottom capacitors when the bias voltage applies and input is zero, respectively; and *g*_4_ and *g*_3_ are the initial large gaps of top and bottom capacitors when the bias voltage applies and input is zero, respectively. 

The turn-on drift generally mentions the shift of zero-offset over time, therefore, the condition of *x* = 0 is considered only and the zero-offset can be expressed as:

(3) $$V_{out}=\frac{e\left(n^{2}-1\right)A_{c}V_{a}}{2g\left(n^{2}+1\right)}$$

where the parameters meet the following relationships:

(4) $$\left\{ \begin{array}{l} g_{2}=g,\;g_{1}=g+e\\ g_{3}=g_{0},\;g_{4}=g_{0}+e\\ g_{0}=ng\;\left(n>1\right)\\ g_{0}>g\gg e \end{array}\right.$$

where *e* represents the gap difference of initial equilibrium state which is determined by the following equation:

(5) $$F_{k}=kx_{0}=F_{et}-F_{eb}\approx \frac{\varepsilon_{0}\varepsilon_{r}A{\left(V_{bias}-V_{f}\right)}^{2}}{g^{2}}-\frac{\varepsilon_{0}\varepsilon_{r}A{\left(V_{f}+V_{bias}\right)}^{2}}{{\left(g+e\right)}^{2}}$$

where *F_k_* is the restoring force formed by two folded beams; *k* is the total stiffness of folded beams; *x*_0_ is the deflection of folded beams along the sensitive axis; *F_et_* and *F_eb_* are the electrostatic forces generated by top and bottom capacitors, respectively [26]; *V_bias_* is the applied bias voltage, *V_bias_* = *V_d_* + *V_a_*sin*ωt*, where *V_d_* and *V_a_* are the DC voltage and amplitude of the AC voltage, respectively; and *V_f_* is the feedback voltage applied on mass block, *V_f_* = *V_out_*.

**Figure 3:**
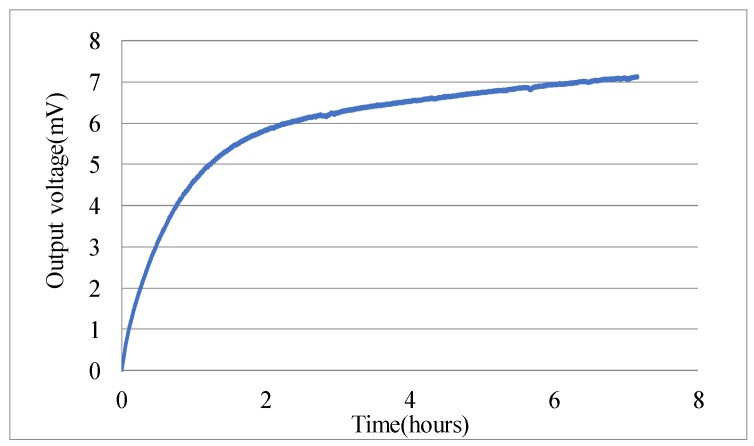
Turning-on drift of one accelerometer.

There exists one and only one steady-state solution of *e* in Equation (5), therefore, the output of accelerometer is a constant if no physical quantities changes and no external distribution applies. However, the experiments of micro-accelerometers showed a continuous change of the output voltage after turning-on, and one example is depicted in Figure 3. Such a long-time drift, about three hours, could not be induced by thermal effects or stress releasing behaviors but resulted from the charging mechanism of dielectric materials.

## 3. Polarization Process

**Figure 4:**
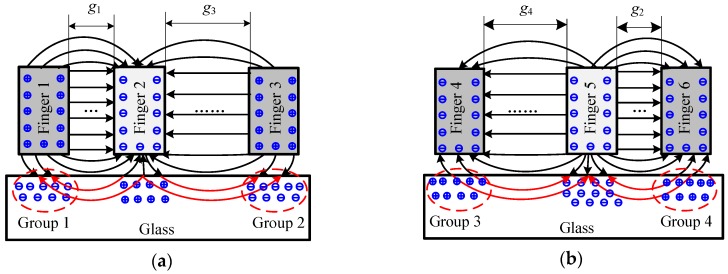
Glass polarization mechanism under an applied DC bias: (**a**) polarization of the top capacitor; and (**b**) polarization of bottom capacitor.

**Figure 5:**
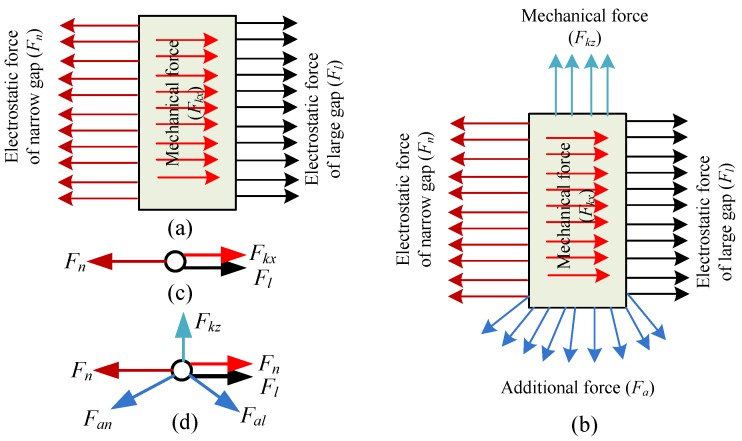
The force acting on finger: (**a**) initial state; (**b**) polarization state; (**c**) simplification of the initial state; and (**d**) simplification of the polarization state.

The dielectric charging occurred in the dielectric materials placed into electric field. The glass of the micro-accelerometer was a structural component used for supporting the silicon die and providing a base for the sputtered gold wire and pads to the electrical connection. In the closed-loop configuration, the feedback voltage, together with the bias DC voltage on the top and bottom electrodes, generated an electrostatic force to rebalance the initial force by pulling back the mass block to the middle position at which the differential capacitances of capacitors were almost equal to zero. The presence of a DC voltage on the electrodes inevitably induced a space charge polarization of the glass substrate, but how the redistribution impacts the sensitive components was related to the specific structural layout of the micro-accelerometer die chip. The polarized results were illustrated by the re-distributed charges close to the surface of the glass in Figure 4. The top electrodes (with gaps *g*_1_ and *g*_3_) with a positive DC voltage attracted the negative ions, while the bottom electrodes (with gaps *g*_2_ and *g*_4_) with negative voltage gathered positive ions. The voltages on the electrodes of the same anchor possessed the same polarity. The level of polarization depended on electrical field intensity determined by both the voltage and the distance between the polarization point and the electrode. Due to the asymmetrical layout of Finger 1 (or Finger 4) and Finger 3 (or Finger 6), the extra electrostatic forces on movable electrode (Finger 2 or Finger 5) from polarized charges Group 1 (or Group 3) and Group 2 (or Group 4) differed from each other. Figure 5 depicted the details of the force change of a single finger on a mass block from an initial state and polarization state. In the initial state, the glass had no polarization and the equilibrium of finger was sustained by the in-plane mechanical force (*F_kx_*) and electrostatic forces of capacitors (*F_n_* and *F_l_*) (the model of distributed load is represented by Figure 5a and the simplified model by Figure 5c). In the polarization state, the additional electrostatic field formed by the redistributed charges would generate additional forces applied to the finger, which disturbed the existed equilibrium and induced both in-plane and out-of-plane movement of finger, therefore, the balance now maintained by the both in-plane and out-of-plane mechanical forces (*F_kx_* and *F_kz_*), electrostatic forces of sensing capacitors (*F_n_* and *F_l_*), and additional force (*F_a_*) (in Figure 5b). Figure 5d shows the simplified model with loads. *F_an_* and *F_al_* represented the additional electrostatic force formed by charges Group 1 and Group 2 in Figure 4, respectively, which caused the mass block moving to the closest electrodes (Figure 1). This movement gave rise to the shift of sensors output. Therefore, the slow movement induced by the slow polarization process leads to a continuous change of output as a drift phenomenon.

## 4. Experiment and Discussion 

**Figure 6:**
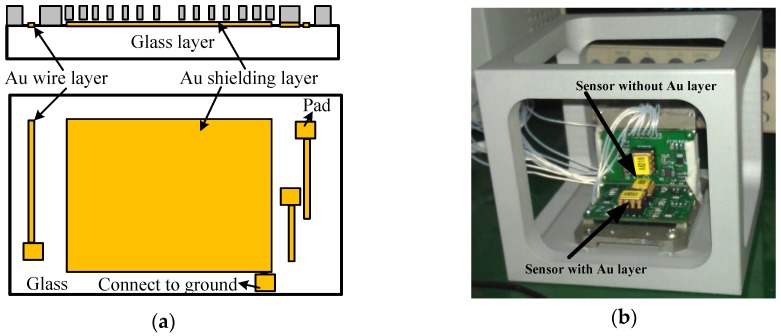
Shielding layer and test platform: (**a**) layout of shielding layer; and (**b**) sensors on platform.

The turn-on drift of micro-accelerometers resulted from the additional electrostatic field formed by the glass polarization, which is very difficult of measure due to the structural complexity and size effects. Although the charge distribution in the glass surface can be measured by a certain KPFM (Kelvin probe force microscopy) method [27,28], it is very difficult to create the same distribution state as the polarized glass of micro-accelerometers. Therefore, the indirect method used in micro-mirrors and MEMS capacitors was used to verify the impacts of polarization. The additional electrical field disturbing the equilibrium was generated from the accumulated charges underneath the sensitive components under the action of the bias DC voltage, so its influence can be eliminated by shielding the glass or conducting the polarized charges to the ground. Figure 6a showed the solution to the drift induced by glass polarization. A gold (Au) layer was fabricated on the glass surface between the substrate and the sensitive structure. The fabrication steps were the same as that in [26] except an extra layer (shielding layer) was deposited together with the wires and pads in the step of sputtering, so the formed electrostatic field was shielded and could not generate additional forces acting on the fingers. Additionally, the shielding layer was also connecting to the ground by a pad and the polarized charges would be conducted to ground. For comparison, both accelerometers with and without Au layers were fabricated on the same silicon wafer to carry out the experiments.

**Figure 7:**
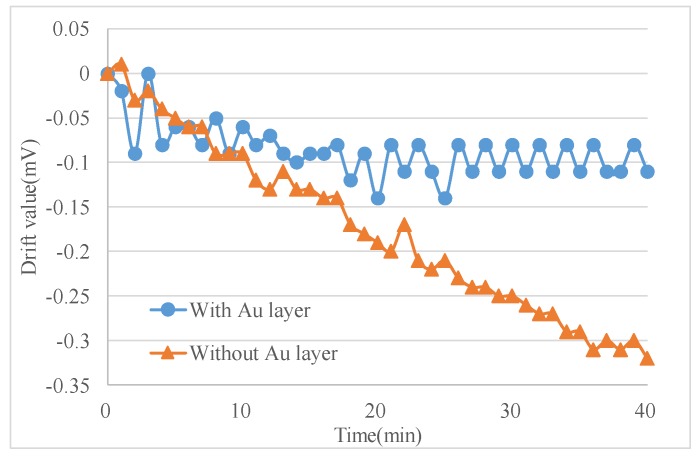
Comparison of sensors with and without an Au layer.

**Figure 8:**
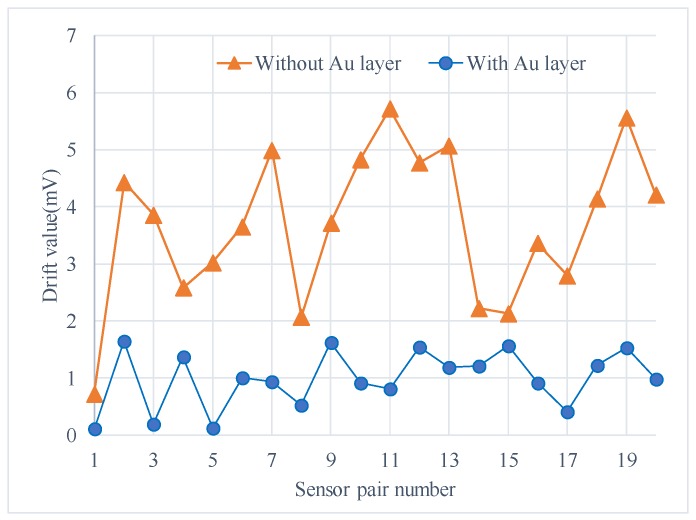
Reduced magnitude of drift for tested sensors with an Au layer as compared with no Au layer.

The experiments were conducted in an incubator and the output signal was collected by RTE1024 R and S oscilloscope (Muenchen, Germany). Each test included two sensors with and without Au layers for a side-by-side comparison (Figure 6b). The signal data were recorded from time of turning-on of the micro-accelerometers to when the output voltage was stable, and the environment temperature was kept constant in an incubator. The output voltage change, the difference between the initial output and the real-time output, was recorded every minute as the drift amount of the accelerometers. Figure 7 listed the comparison of two selected sensors. The one with the Au layer exhibited about a 0.1 mV drift with a very small drift period, while the one without the Au layer exhibited a 0.35 mV drift in 40 min and showed no stable output. It was obviously the Au-shielding layer that greatly decreased the drift of micro-accelerometers both in the output change and settling time. Figure 8 showed the experiment results of 20 pairs of micro-accelerometers after both acquiring a stable output voltage. It was indicated that the average magnitude of drift decreased from 3.69 to 0.99 mV because the glass polarization-induced drift had been eliminated by the shielding layer, which led the polarized charges to the ground and no additional electrostatic field formed to disturb the current balance. Nevertheless, the remaining drift existing in the tests was related to the charge movement of other components [12].

## 5. Conclusions

The glass polarization effect induced by the DC bias voltage which was used to form a feedback force in the closed-loop system was considered as one of the main causes of turn-on drift of micro-accelerometers in this paper. The drift phenomenon was due to the asymmetrical actions generated from the additional electrostatic forces formed by polarized electric fields, resulting from the asymmetry of the structure layout. The experiments on sensors with Au layers and without Au layers clearly demonstrated that the glass polarization-induced drift could be suppressed by shielding the extra fields created by the accumulated charges, and the average drift magnitude of shielding sensors was about 27% of the previous ones. The future work, based on the current outcomes, is to study the effects of feedback voltage of Equation (5) on the glass polarization when the input acceleration is not zero and to model the polarization behaviors under different temperature.
